# Athermally photoreduced graphene oxides for three-dimensional holographic images

**DOI:** 10.1038/ncomms7984

**Published:** 2015-04-22

**Authors:** Xiangping Li, Haoran Ren, Xi Chen, Juan Liu, Qin Li, Chengmingyue Li, Gaolei Xue, Jia Jia, Liangcai Cao, Amit Sahu, Bin Hu, Yongtian Wang, Guofan Jin, Min Gu

**Affiliations:** 1 Centre for Micro-Photonics, Faculty of Science, Engineering and Technology, Swinburne University of Technology, Hawthorn, Victoria 3122, Australia; 2 Beijing Engineering Research Center for Mixed Reality and Advanced Display, School of Optoelectronics, Beijing Institute of Technology, Beijing 100081, China; 3 Environmental Engineering & Queensland Micro- and Nanotechnology Centre, Griffith University, Brisbane, Queensland 4111, Australia; 4 State Key Laboratory of Precision Measurement Technology and Instruments, Tsinghua University, Beijing 100084, China

## Abstract

The emerging graphene-based material, an atomic layer of aromatic carbon atoms with exceptional electronic and optical properties, has offered unprecedented prospects for developing flat two-dimensional displaying systems. Here, we show that reduced graphene oxide enabled write-once holograms for wide-angle and full-colour three-dimensional images. This is achieved through the discovery of subwavelength-scale multilevel optical index modulation of athermally reduced graphene oxides by a single femtosecond pulsed beam. This new feature allows for static three-dimensional holographic images with a wide viewing angle up to 52 degrees. In addition, the spectrally flat optical index modulation in reduced graphene oxides enables wavelength-multiplexed holograms for full-colour images. The large and polarization-insensitive phase modulation over *π* in reduced graphene oxide composites enables to restore vectorial wavefronts of polarization discernible images through the vectorial diffraction of a reconstruction beam. Therefore, our technique can be leveraged to achieve compact and versatile holographic components for controlling light.

## Abstract (web summary)

Owing to its electronic and optical properties, graphene holds potential for flat display systems. Here, Li *et al*. write wide-angle, full-colour, three-dimensional holographic images using subwavelength, multilevel index modulation of athermally reduced graphene oxide by a single femtosecond pulse.

## Graphical abstract

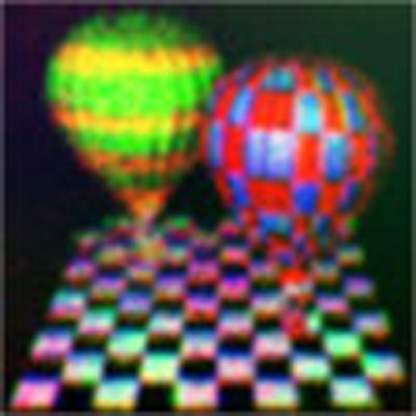

Holography^1^ offers a way to reconstruct wavefronts of light with both amplitude and phase information for real three-dimensional (3D) images. The physical realization of wide-angle 3D holographic images relies on the generation of subwavelength-scale refractive-index/phase modulation according to the holographic correlation. As such, there are soaring developments of directly fabricating subwavelength-scale phase modulations in a variety of holographic materials through both reversible and write-once methods^2^,^3^,^4^,^5^,^6^,^7^. Even though updatable 3D holographic images have been demonstrated in photorefractive polymers through the two-beam interference with the assistance of a high voltage^2^,^3^, multiview stereoscopic approaches have to be implemented to compensate the limited viewing angle of each perspective image or hologram, which has micrometre-scale refractive-index modulations associated with the non-locality of the polymeric photorefractivity^8^. Removing the complexity, single-beam computer-generated holography^9^,^10^ combined with the recent advance in metamaterials^4^ and metasurfaces^5^,^6^ has enabled write-once subwavelength-scale phase manipulations for 3D holographic images with the potential of wide viewing angles. In addition to the low resolution (less than 800 pixels in each direction) incapable of practical applications, however, the resonance nature in these metallic nanostructures inherently hampers the reconstruction of full-colour and polarization-dependent wavefronts of light^4^,^5^,^6^.

On the other hand, graphene^11^, a two-dimensional carbon material with extraordinary electronic and optical properties, has offered a bottom-up new material platform for next-generation nanophotonic devices^12^,^13^,^14^,^15^,^16^. In this context, graphene oxide (GO) covalently bonded with oxygen-containing functional groups becoming an electronic and optical active material once it is reduced back towards graphene^17^, has emerged as a straightforward approach to directly writing nanoscale contrast in the material permittivity and hence for patterning reduced graphene-oxide (rGO)-based devices including supercapacitors^18^,^19^,^20^, flexible circuits^21^, sensors^22^,^23^, nanomechanical resonators^24^,^25^ and solar cells^26^,^27^. The structure of rGO is analogous to graphene but with some residual oxygen and structural defects, yielding different optical and electronic properties^28^ and opens the potential for write-once holograms for wide-angle and full-colour 3D images.

So far, controlling the extent of the localized reduction of GOs has been investigated by a number of thermal treatment approaches including near-field scanning hot tips^29^,^30^ and continuous-wave- or quasi-continuous pulsed laser irradiations^19^,^20^. In those configurations, a high-temperature deoxygenation process through the constant thermal treatment is essential to rGOs and produce a contrast in the conductivity (*σ*(*ω*)) to vitalize their electronic properties. In addition to the difficulty to obtain the optimal contrast at high temperatures close to 1,000 °C^26^, it is elusive to confine the heat diffusion and generate a localized temperature field for the subwavelength-scale reduction.

In this report, we discover a subwavelength-scale contrast in refractive index through an athermal photoreduction process induced by a single femtosecond (fs) pulse. Therefore, the tunable extent of the athermal photoreduction allows for writing the phase modulation according to the holographic correlation for 3D-wide viewing-angle images.

## Results

### Athermally photoreduced GOs

**Figure 1:**
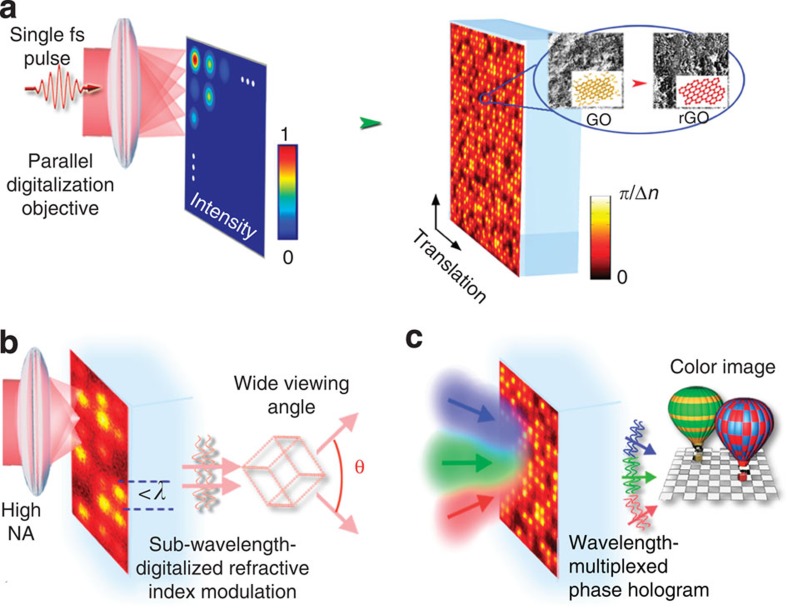
rGO holograms by a single femtosecond pulse for 3D images with wide viewing angles and colour images. (**a**) Schematic illustration of the optical digitalization of refractive-index/phase modulation by the athermal photoreduction using a single fs pulse. The subwavelength-scale phase modulation from 0 to π is finely tuned by the intensity of a fs beam. The area-by-area parallel digitalization is achieved through an objective capable of generating MMAs with variant intensities in each focal spot corresponding to the phase correlation. (**b**) Scheme of wide-angle 3D images by confining the photoreduction at a subwavelength scale through increasing the NA of the parallel digitalization objective. (**c**) Reconstruction of colour objects through the wavelength-multiplexed phase hologram recorded in GO polymers.

We record holographically correlated refractive-index modulation in the GO-dispersed photopolymers through the area-by-area digitalization by multilevel multifocal arrays created by the Debye diffraction method^31^, as shown in Fig. 1a, while the intensity of each focal spot produced by this method and hence the refractive-index modulation in the rGO composite can be finely tuned in terms of the correlation. By removing the undesired reduction associated with the diffusion of accumulative heating, the athermal photoreduction through a single fs pulse is confined to a diffraction-limited region of each focal spot. Consequently, increasing the numerical aperture (NA) of the objective used for parallel digitalization can lead to a decreased size of each focal spot down to a subwavelength scale of the reconstruction beam, and hence an increased viewing angle, as illustrated in Fig. 1b. Moreover, the spectrally flat refractive index of the rGO composite in the visible range^32^,^33^ makes it ideal for multicolour holography. A wavelength-multiplexed phase hologram can be used for colour images, where light waves at three wavelengths are incident simultaneously in a tilted configuration to synthesize corresponding colour components (Fig. 1c).

**Figure 2:**
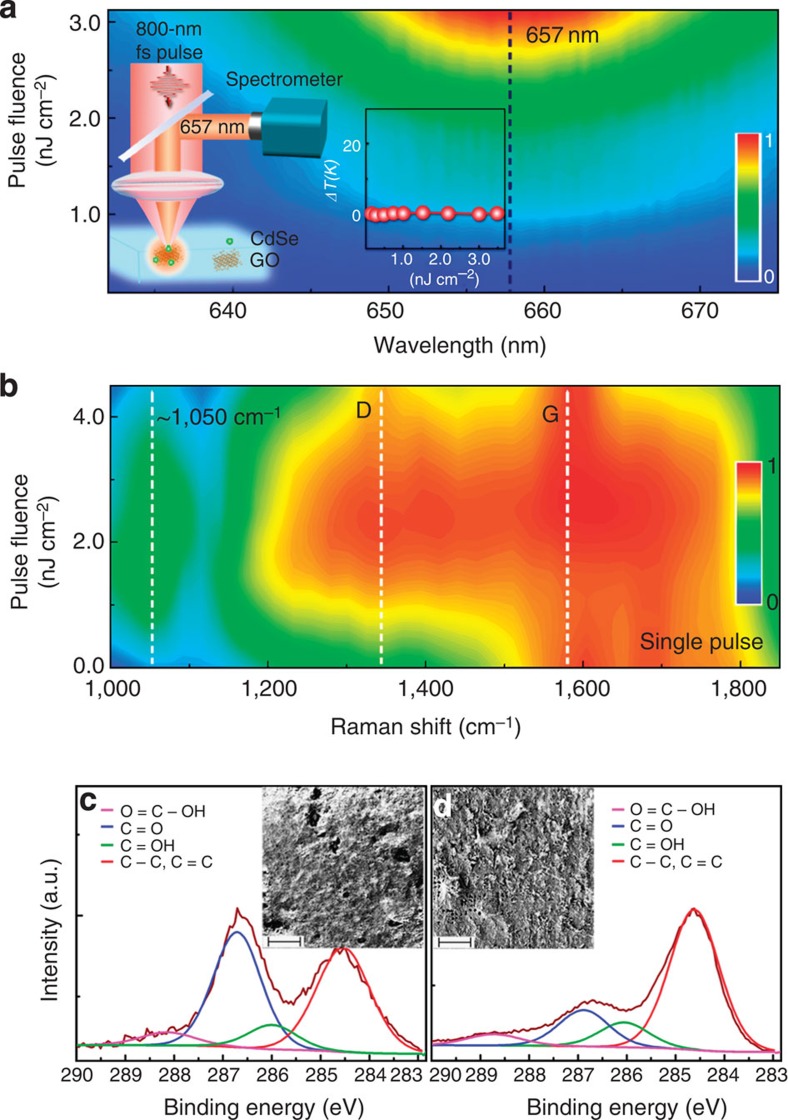
Athermal photoreduction of GOs by a single fs pulse. (**a**) Spectral shift of the two-photon fluorescence of CdSe nanoparticles irradiated by single fs pulses at a variety of pulse energy densities. The dashed line indicates the peak position of the CdSe fluorescence spectra. The insets show the experimental configuration for the focal temperature measurement and extracted temperature increment. (**b**) Raman spectra of the rGO polymers as a function of the fs pulse fluence. Three dashed lines indicate the D-bands, G-bands and 1,050 cm^−1^, respectively. X-ray photoemission spectroscopy spectra of GOs before (**c**) and after (**d**) photoreduction by a single fs pulse at the pulse fluence of 2 nJ cm^−2^. Insets are the scanning electron microscope (SEM) images of GOs before (**c**) and after (**d**) the photoreduction.

The photoreduction of as prepared GO polymers by a single fs pulse (Methods) into rGO polymers was verified by micro-Raman spectroscopy and X-ray photoemission spectroscopy in Fig. 2. The accumulative heating can be excluded from the experiment since a single pulse is employed. The athermal nature of the observed photoreduction was confirmed by monitoring the temperature increment in the focal region using fluorescent CdSe spherical nanoparticles as nanothermometers^34^,^35^. The inset of Fig. 2a shows the experimental set-up for the temperature measurement by monitoring the spectral shift of the fluorescence peak of CdSe nanoparticles (Methods). Indeed, no notable spectral shift of CdSe nanoparticles (Fig. 2a) and hence no temperature increment (inset of Fig. 2a) were observed at the entire range of the pulse fluence used for the photoreduction. Figure 2b depicts the Raman spectra of rGOs at different fluences of the single pulse irradiation. The characteristic D- and G-bands centred at 1,348 and 1,578 cm^−1^, respectively, are broad and accompanied by a peak at 1,050 cm^−1^, which is associated with the vibration bands of carbon atoms at the presence of oxygen-containing groups^36^. The strength of the peak at 1,050 cm^−1^ is decreased as the pulse fluence increases, indicating a deoxygenation process. The intensity ratio between D- and G- bands remains almost constant during the photoreduction accompanied by the rising of the 2D bands (Supplementary Fig. 1). The observed photoreduction can be possibly attributed to the photoionization after absorbing the pulse (Supplementary Discussion).

The strength of reduction can be controlled by the fluence of the single fs pulse, which can be quantified by X-ray photoemission spectroscopy. Figure 2c,d shows the photoemission spectra of the carbon 1s of GOs before and after the photoreduction. The deoxygenation through the athermal photoreduction process is evident by a drastic decrease of the C–O–C (286 eV), C=O (287 eV) and OH–C=O (288.5 eV) peaks, meaning a restoration of the pure C–C/C=C bond from 28 to 54%. The deoxygenation was accompanied by a morphology change from the disordered manner in GO stacks to micro-sized rGO flakes (insets of Fig. 2c,d). In contrast, quasi-continuous pulsed irradiation operated at a laser repetition rate of 80 MHz induces significant accumulative heating and a pronounced temperature increment in the focal plane (Supplementary Figs 2 and 3). The deoxygenation by the constant thermal treatment changed the morphology to micrometre-long rGO wrinkles (Supplementary Fig. 4).

### Reduced graphene-oxide holograms for 3D images

**Figure 3:**
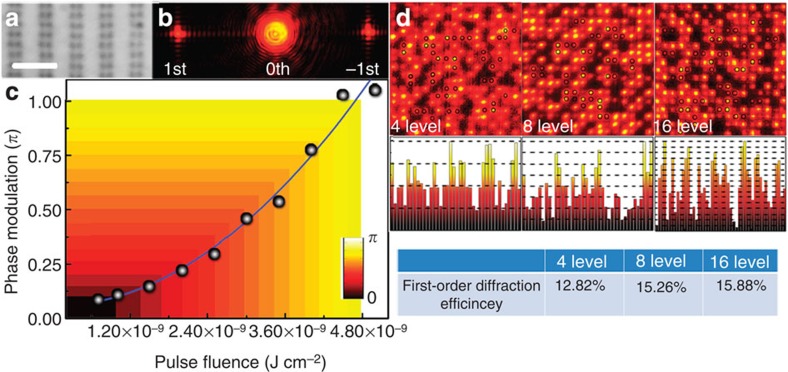
Localized phase modulation produced by the refractive-index change of GOs through the tunable extent of the photoreduction. (**a**) Wide-field image of one typical phase grating composed of arrays of refractive-index modulation pixels through the athermal photoreduction with a parallel digitalization objective. Scale bar, 6 μm. (**b**) Example of diffraction images of ±1st and 0th orders. (**c**) Phase modulation strength as a function of the pulse fluence of the fs beam. The circles are experimental data and the blue curve is the guide for eyes. The colour levels indicate digitalized phase modulations by judicious control of the intensity of the fs beam. (**d**) Microscopic images of a section of a rGO hologram through 4-, 8- and 16-level digitalized photoreduction processes of GO polymers and their statistics of strengths of randomly selected refractive-index pixels. The table shows the comparison of the first-order diffraction efficiency of rGO holograms with different levels of modulation.

The athermally digitalized photoreduction of GOs in photopolymers enables one to judiciously control the refractive index of rGOs as verified by the diffraction experiment in Fig. 3, which is crucial for the subsequent recording of the correlated phase modulation in individual pixels for rGO holograms. Figure 3a–c depicts the measured phase accumulation when the reconstruction beam propagates through the rGO polymer irradiated by the single fs pulse (Methods). The wide-field image of one typical example of the phase grating composed of arrays of refractive-index pixels through the photoreduction and its diffraction image are shown in Fig. 3a,b, respectively. Intensifying the strength of the photoreduction by increasing the pulse fluence at each focal spot leads to an exponential increase in the strength of the phase modulation, which is a basis to finely digitalize the localized phase modulation by judiciously configuring the exposing intensity (Fig. 3c). The large dynamic range of the phase modulation to *π* opens the possibility of multilevel rGO holograms with a high diffraction efficiency (Methods). Figure 3d shows examples of microscopic images of 4-, 8- and 16-level digitalized rGO holograms. Increased first-order diffraction efficiency was observed as increasing the levels of modulations. A diffraction efficiency of 15.88% was achieved at a 16-level modulation.

**Figure 4:**
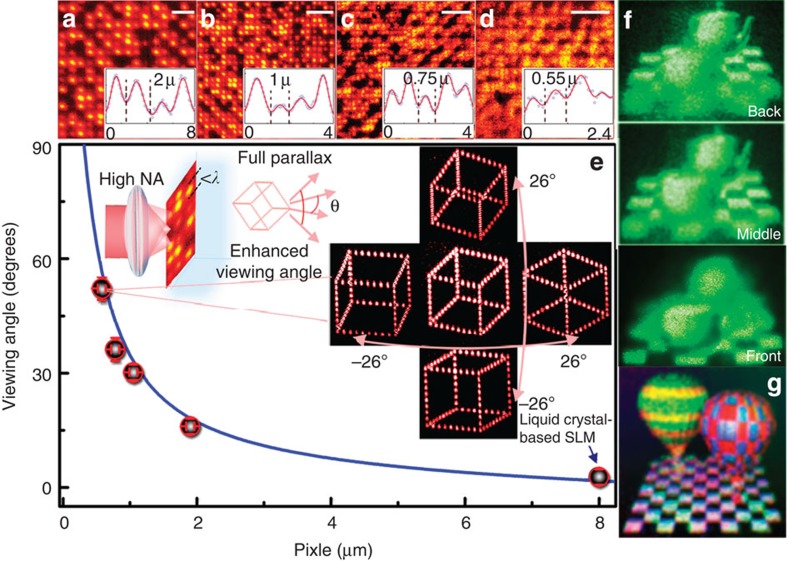
rGO holograms for 3D colour images. Typical examples of microscopic images of sections of rGO holograms produced by objectives with different values of the NA. Scale bars, 5 μm. The insets show examples of intensity cross-sections of constitutive pixels with an effective size of 2 μm (**a**), 1 μm (**b**), 0.75 μm (**c**) and 0.55 μm (**d**). (**e**) Viewing angle as a function of the size of the constitutive pixel. The circles are experimental data and the blue curve presents calculation results. The inset shows reconstructed images of a rGO hologram with a pixel size of 0.55 μm captured at different viewing angles. The liquid crystal-based spatial light modulator (SLM, indicated by the arrow) with a pixel size of 8 μm has a limited viewing angle of ∼5 degrees. (**f**) Images of reconstructed 3D objects, two teapots, captured at different depths. (**g**) Reconstruction of colour objects, two balloons through a wavelength-multiplexed phase hologram recorded in GO polymers.

A computed-generated 3D cubic was obtained by the point source method^37^ and fast digitalized into a phase profile through translating the GO polymer sample with respect to the focal plane of the parallel digitalization objective (Supplementary Fig. 5; Supplementary Movie 1). Since the athermal photoreduction can be confined within the diffraction-limited focal voxel of the multilevel multifocal array, we can increase the NA of the parallel digitalization objective to reduce the constitutive pixel size below the wavelength of the beam employed for the image reconstruction, which is impossible when a thermal reduction induced by quasi-continuous pulsed irradiations is employed (Supplementary Figs 2 and 3). Examples of microscopic images of a section of the holograms recorded by different NA are shown in Fig. 4a–d. Figure 4e depicts that the viewing angle is drastically increased by reducing the constitutive pixel size, which is reasonably consistent with the calculation (Methods). In particular, when the pixel size is reduced to 0.55 μm, the viewing angle can be increased up to 52 degrees (inset of Fig. 4e; Supplementary Movie 2), which is one order of magnitude larger than that of metamaterial^4^,^5^,^6^ or carbon nanotube^7^ holograms with a similar image size. For comparison, a commercially available liquid crystal-based spatial light modulator (SLM) (1,920 × 1,024 pixels and pixel size of 8 μm) with a viewing angle of ∼5 degrees is also shown. Since the space–bandwidth product^38^ of a hologram is approximately proportional to the product between the number of constitutive pixels in each direction, the rGO hologram exhibits the remarkably extended space–bandwidth product through the parallel digitalization, by one order of magnitude larger than that of metasurface- and metamaterial-based write-once holograms^4^,^5^,^6^. Thus, the reconstruction of 3D images with high-resolution full-depth perceptions is feasible (Methods). Figure 4f shows images of reconstructed objects, two teapots floating above the rGO hologram, captured at different depths (Supplementary Movie 3). The rGO holograms can also be up-scalable for practical applications with sufficient high resolution (Supplementary Fig. 6).

In addition, the spectrally flat refractive-index modulation of the photoreduction process (Supplementary Fig. 7) enables its application in colour 3D images. Full-colour images can be synthesized by a wavelength-multiplexed phase hologram with angular offsets at corresponding wavelengths. For reconstruction, three laser beams at the wavelengths of 405, 532 and 632 nm, respectively, were employed in a tilted configuration (Fig. 1c). The white colour balance was obtained by carefully controlling the powers of the three constitutive laser beams. Reconstruction of full-colour objects, two balloons, can be seen vitally from the multiplexed rGO hologram (Fig. 4g; Supplementary Movie 4).

**Figure 5:**
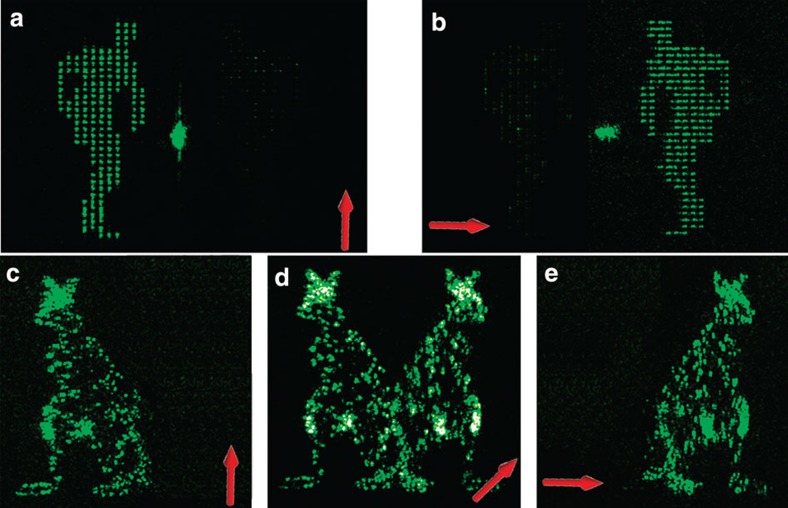
Vectorial holographic reconstruction of polarization discernible images. The reconstructed vectorial wavefront of two human figures can be discerned at the vertical (**a**) and horizontal (**b**) polarization angles, respectively. The reconstructed 3D vectorial wavefront, two kangaroos with different polarization orientations, can be discerned at the vertical polarization angle (**c**), viewed simultaneously at 45 degrees (**d**) and discerned at the horizontal polarization angle (**e**), respectively. The arrows indicate the polarization alignment angles of the analyzer.

To date, the reconstruction of vectorial or polarization-dependent wavefronts, termed as vectorial holographic images where polarization orientations on a 3D wavefront are spatially variant, still remains elusive for two reasons. First of all, a large modulation strength over *π* in constitutive pixels of a pure phase hologram to generate the constructive or deconstructive interference of diffracted fields is crucial to realize spatially varying polarization orientations through the vectorial diffraction^39^,^40^. Meanwhile, the isotropic or polarization-insensitive refractive-index modulation in each pixel is essential to record polarization-multiplexed phase holograms without any distortion of the vectorial field distribution. In this context, rGO composites by the athermal photoreduction provide the perfect material platform to fulfil the requirement for the reconstruction of vectorial wavefronts. Such reconstructed vectorial wavefronts carrying the polarization-sensitive information of objects can be distinctively discerned by rotating the polarization angle of the analyzer (Supplementary Fig. 8). As an example, Fig. 5a,b shows the captured images of the reconstructed vectorial wavefront of two human figures with left and right parts clearly discerned at the vertical and horizontal polarization angles, respectively. The two images carried by the wavefront are smoothly transitioned from one to the other when rotating the polarization alignment of the analyzer (Supplementary Movie 5). Figure 5c–e shows that the reconstructed 3D vectorial wavefront, two kangaroos with different polarization orientations, can be discerned at corresponding polarization angles or viewed simultaneously.

## Discussion

The generation of multilevel modulations in the refractive index of rGOs, which does not require any solvents or post-processing, holds the potential for *in situ* fabricating rGO-based electro-optic devices. The athermal nature of the photoreduction by a single fs pulse enables its applications in a variety of temperature-sensitive conditions. The write-once rGO holograms through the subwavelength-scale and tunable photoreduction of GOs could be implemented in multiple applications to achieve compact and versatile holographic components for controlling light. On the other hand, updatable holographic components by SLMs are highly demanded for applications requiring *in situ* and dynamic reshaping of the wavefront of light. The large size of phase pixels on the order of tens of wavelengths restricts their applications in compact optics and micrometre-sized beam engineering, even though their viewing angles could be extended through SLM array techniques^41^ and acousto-optic modulators combined with mechanical scanners^42^,^43^,^44^. Nevertheless, the reversible reduction and oxidation of GOs by switching the polarity of electrical stimulus^45^ might be analogously obtained through the precise control of the laser irradiance at the presence of corresponding photocarrier generators, and hence for updatable rGO holograms. This effort will lead to unquestionably potential for rGO holograms in versatile applications in optical data storage, information processing and imaging.

## Methods

### Preparation of GO polymers

GOs were prepared with a well-established recipe by the chemical oxidation of natural graphite followed by the exfoliation using sonication for 2 h in water^46^. Unexfoliated particles were removed by centrifugation at 12,000 r.p.m. for 20 min. The brown supernatant containing ∼3 mg ml^−1^ of GOs was obtained. The GO solution was mixed with poly(vinyl alcohol) water solution with a concentration of 20 wt% at a volume ratio of 1 to 5. To prepare GO-polymer thin films, the mixture was spin coated on a cover glass and dried at room temperature. For the scanning electron microscope image, the pure GO solution was spin coated without poly(vinyl alcohol).

### Focal temperature measurement

CdSe spherical nanoparticles of 4 nm in diameter from Invitrogen Inc. were dispersed in GO-polymer samples at a concentration of 1 μM. The peak (∼657 nm) shifts of their two-photon fluorescence spectra under single fs pulse illumination at a variety of fluences were monitored by a charge-coupled device for measuring the temperature increase in the focal region. The temperature increase was extracted by a linear dependence of ∼0.1 nm per °C^34^,^35^.

### Photoreduction system

A fs-pulsed laser beam at the wavelength of 800 nm (a repetition rate of 1 KHz and a pulse width of 100 fs) was employed for the photoreduction. The laser beam was focused by an objective lens with the NA of 1.2 down to a lateral size of 0.55 μm in the focal area. The patterned photoreduction was realized by laterally translating the sample across the focal plane.

### Characterization of the phase modulation strength

Refractive-index gratings with a period of 4 μm and a duty cycle of 1/2 were recorded into the sample. The ratio of the first-order to the zero-order diffraction intensities (*R*) was measured to characterize the phase modulation strength following^47^


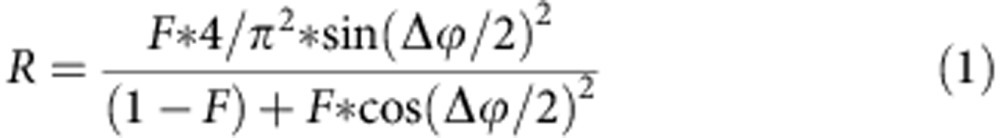


where *F* is the filling factor determined by the ratio of the grating area to the beam size and Δ*ϕ* is the phase modulation strength.

### Diffraction efficiency of multilevel phase holograms

The diffraction efficiency of multilevel phase holograms can be simplified as the desired continuous phase profile minus an error phase profile. Then the first-order diffraction efficiency of such a multilevel phase hologram can be given as^48^


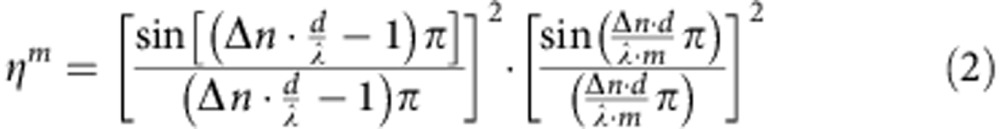


where *m* is the level of the phase modulation, Δ*n* is the refractive-index modulation and *d* is the thickness of the focal depth. It reveals that an increased diffraction efficiency can be achieved through increasing the modulation levels.

### Viewing angles of rGO 3D images

The maximal diffraction angle for a rGO hologram with round pixels is given as


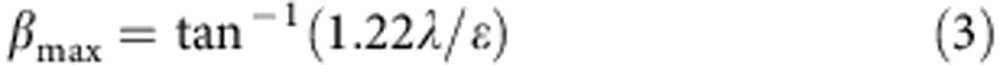


where *λ* is the wavelength and *ɛ* is the spatial frequency of the fringe with the largest value of two times the pixel size *p*. The maximal size of the reconstructed object is given as


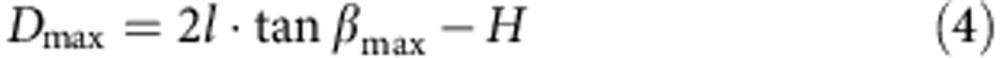


where *l* is the distance of the reconstructed image with respect to the rGO hologram and *H* is the size of the hologram. Therefore, the viewing angle can be expressed as


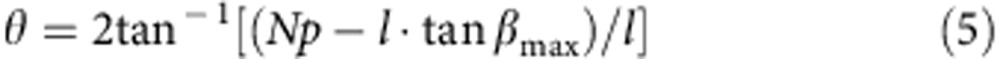


where *N* is the number of pixels. From the above equations, it can be seen that the viewing angle is drastically increased as the size of constitutive pixels decreases.

### Space–bandwidth product and resolution of holographic images

The space–bandwidth product of a hologram can be expressed as^38^


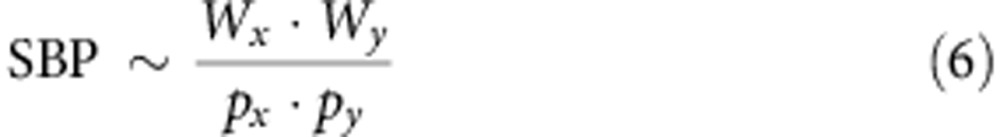


where *W*_*x*/*y*_ and *p*_*x*/*y*_ are the width and pixel size of the hologram in the *x*- and *y*-direction, respectively. It can be approximated to the product between the number of pixels in each direction. For comparison, typical rGO holograms in the experiment with 4,000 × 4,000 pixels exhibit a space–bandwidth product of one order of magnitude larger than that of the state-of-the-art holograms composed of subwavelength-scale phase modulations such as metasurface (800 × 800 pixels)^5^ and carbon nanotube holograms (300 × 300 pixels)^7^.

The resolution of the reconstructed image is defined as^49^,


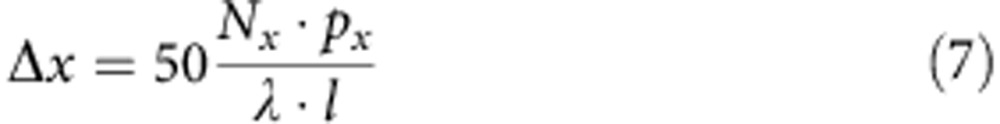


where *l* is the distance of the reconstructed image with respect to the rGO hologram plane and *N*_*x*_ and *p*_*x*_ are the number of pixels and the pixel size in the *x*-direction of the rGO hologram, respectively. At a reconstruction distance of 300 mm, one example of rGO holograms with 4,000 pixels and a pixel size of 2 μm enables an image resolution more than 2,500 d.p.i., corresponding to one order of magnitude better than the reported metasurface^5^ and carbon nanotube holograms^7^ with a similar image size.

## Author contributions

X.L. and M.G. proposed the idea and the strategy for experimental design and data analysis and completed the writing of the paper. X.L. completed the optical digitalization and holographic image characterization. H.R. contributed to the algorithms of the vectorial hologram. X.C. contributed to the material synthesis. J.L., G.X., J.J., B.H. and Y.W. contributed to the view angle calculation and designed the computer generated holograms. Q.L. contributed to the XPS experiment and involved in the discussion. C.L., L.C. and G.J. contributed to the discussion and paper writing. A.S. contributed to the development of the optical digitalization system.

## Additional information

**How to cite this article:** Li, X. *et al*. Athermally photoreduced graphene oxides for three-dimensional holographic images. *Nat. Commun.* 6:6984 doi: 10.1038/ncomms7984 (2015).

## Supplementary Material

Supplementary Information Supplementary Figures 1-8, Supplementary Discussion, Supplementary Methods, Supplementary References.

Supplementary Movie 1 A typical example of the parallel optical digitalization of 3 by 3 areas, with 60 by 60 refractive-index pixels in each area. The sample was laterally translating in the focal plane of a multi-level multifocal array (MMA) composed of 20 by 20 focal-spots to produce the photoreduction of graphene oxide (GO)-polymers.

Supplementary Movie 2 Horizontal and vertical parallax of the 3D cubic captured at different viewing angles by the CCD. The reduced-graphene-oxide (rGO) hologram was recorded through a parallel digitalization objective with a numerical aperture (NA) of 1.2 and a constitutive pixel size of 0.55 μm.

Supplementary Movie 3 3D teapots captured at different viewing depths by the CCD. The rGO hologram was recorded through a parallel digitalization objective with a 20-fold enhanced space-bandwidth product and a 5-fold enhanced resolution in each direction compared with that enabled by the state-of-the-art metamaterial holograms 1-3.

Supplementary Movie 4 Reconstruction of full color objects, two balloons captured at different viewing angles by the CCD. The multiplexed rGO hologram was recorded through a parallel digitalization objective with a total number of pixels of 4000 by 4000.

Supplementary Movie 5 Vectorial holographic reconstruction of polarization discernible images of two human figures captured by the CCD at different polarization angles of the analyzer
