# Supplementary material for: Athermally photoreduced graphene oxides for three-dimensional holographic images
Source: Nat Commun. 2015 Apr 22;6:6984. doi: 10.1038/ncomms7984 (PMC4421811; doi:10.1038/ncomms7984)
Supplement: Supplementary Information — Supplementary Figures 1-8, Supplementary Discussion, Supplementary Methods, Supplementary References. [file ncomms7984-s1.pdf]

Supplementary Figures

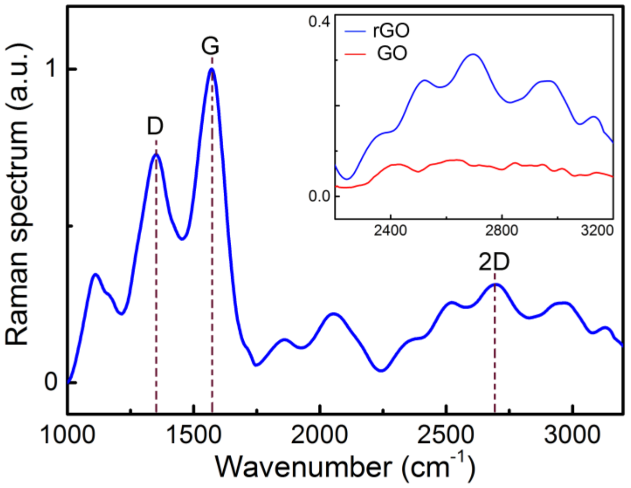

Supplementary Figure 1. Full Raman spectrum of the rGO by a single pulse at the pulse fluence of 2 nJ/cm^2. The inset shows the comparison of the 2D bands of the rGO with the graphene oxide (GO). The rising of the 2D bands centered at 2688 cm^{-1} unambiguously indicates the reduction of GOs to rGOs, although the overall magnitude of the 2D bands compared to the G bands is weak. The 2D bands exhibit a broad distribution with a low intensity, which might be attributed to the multilayer structure of rGOs within the focal voxel or an incomplete reduction with residual oxygen components [4].

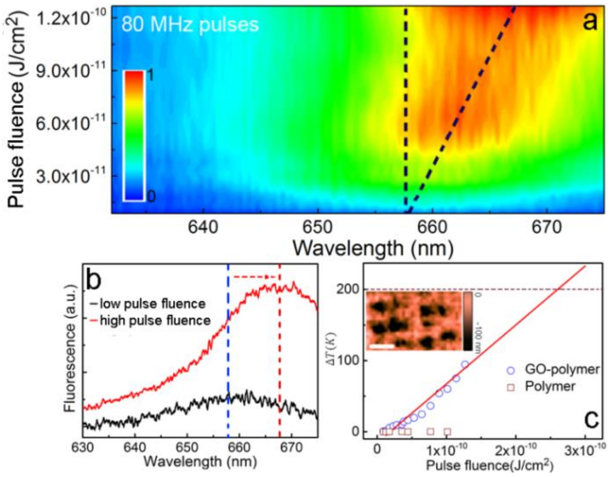

Supplementary Figure 2. a, measured focal temperature increase of GO-polymers by a quasi-continuous pulsed irradiation at a 80 MHz repetition rate as a function of the pulse fluence. The spectral shift of CdSe nanoparticles irradiated at a variety of pulse fluences [5]. The dashed lines indicate peak positions of the fluorescence spectra of CdSe nanoparticles. b, Two-photon fluorescence spectra of CdSe nanoparticles at a low and high pulse fluence, respectively, showing a pronounced spectral shift close to 10 nm. c, Extracted temperature (circles) at different pulse fluences. The focal temperature increase by the 80 MHz pulse trains at a threshold pulse fluence of 0.12 nJ/cm^2 for the photoreduction is $\sim 100^oC$. This temperature threshold is consistent with the previous observation of thermochemical reduction by a constant thermal treatment [6]. No notable focal temperature increment can be observed in the control sample without the GO doping (squares), since there is no linear absorption at the excitation wavelength of 800 nm. The solid line is the fitting assuming that the heat effect is dominantly caused by the linear absorption. The dashed line indicates the temperature where the deformation of the polymer matrix occurs [7]. The inset shows the atomic force microscopic image of the GO-polymer after the photoreduction at the fluence of 0.25 nJ/cm^2. The dark parts represent the written pixels exhibiting the morphologic change in the GO-polymer. Owing to the heat diffusion and the deformation, the size of the

written pixels is significantly larger than the diffraction limited focal region of a high NA objective. The scale bar is 3 μm.

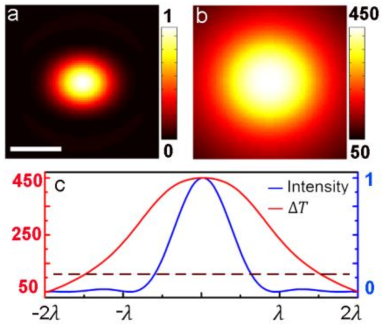

Supplementary Figure 3. The comparison of the intensity distribution (a) and accumulative heating induced temperature increase (b) in the focal plane of an objective with high NA = 1.2 at a moderate laser fluence of 5× 10^{-10} J/cm^2 and an exposure time of 10 ms. c, The comparison of cross section plots between the focal intensity and the temperature rise. The dashed line indicates the temperature threshold ($\sim 100^oC$) for the reduction of GOs. The temperature rise and the subsequent heat dissipation caused by the absorption of successive quasi-continuous fs pulses (80 MHz) are calculated by using the heat conduction equation. The spatial and temporal dependence of the temperature change within the focal region in the sample is given by [8]

$$\nabla T(r,t) = \sum_{n=1}^{m} \frac{E(r_0)}{8k(\frac{k}{\rho c_p})^{\frac{1}{2}}(\pi n t)^{\frac{3}{2}}} \exp(\frac{(r-r_0)^2}{4\frac{k}{\rho c_p}nt}) \quad (1)$$

where $E(r_0)$ is the pulse energy distribution given by the objective, ρ, c_p and k is the density (2000 $kg \cdot m^{-3}$), the heat capacity (1.7 $J \cdot cm^{-3} \cdot K^{-1}$) and the thermal conductivity (4 $W \cdot m^{-1} \cdot K^{-1}$) of GOs [9], respectively, t is the time interval between two successive pulses (12.5 ns for the 80 MHz repetition rate), n is the number of pulses, r_0 is the relative distance. It is clearly revealed that the accumulative heating by the quasi-continuous pulsed beam significantly diffuses out the focal region of the high NA objective and corroborates with the experimental observation of the reduction of GOs over an area with a radius of a few

wavelengths. Thus, the sub-wavelength-scale refractive-index change cannot be achieved with a photothermal reduction process through a quasi-continuous pulsed laser beam.

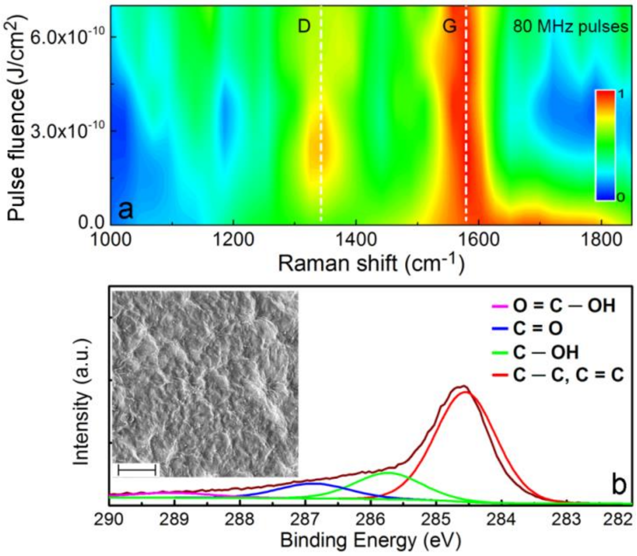

Supplementary Figure 4. a, Raman spectra of rGOs by 80 MHz fs pulse trains as a function of the pulse fluence. b, XPS spectra of rGOs and scanning electron microscope (SEM) image of rGOs showing a morphology of micrometer-long wrinkles. The scale bar is 10 μm.

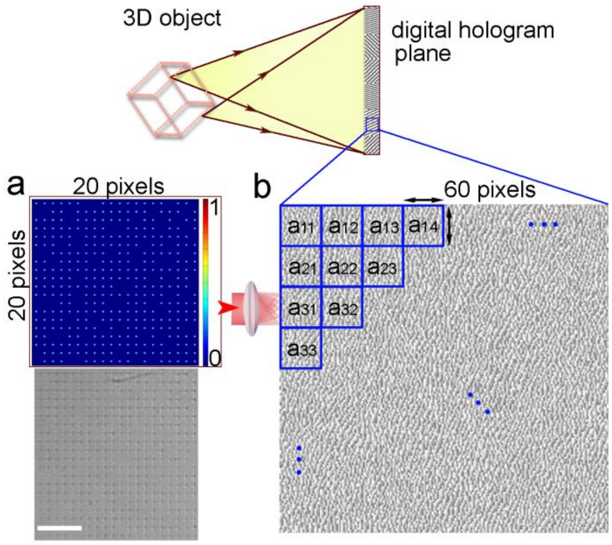

Supplementary Figure 5. a, One typical example of calculated multi-level multifocal arrays (MMAs) composed of 20 by 20 focal-spots with spatially-varying intensities using the Debye-diffraction method [10-12] (see Supplementary Methods) and the wide-field image of the corresponding phase/refractive-index modulation in GO-polymers. The scale bar is 30 μm .b, computer generated holograms of a 3D object by using the point source method [13] and schematic illustration of parallel optical digitalization by dividing the phase profile into arrays of areas consisting of 60 by 60 pixels. Each area of the rGO hologram is recorded by laterally translating the GO-polymer sample in the focal plane of the MMA. A typical example of writing rGO holograms of 3 by 3 areas is shown in the Supplementary Movie 1.

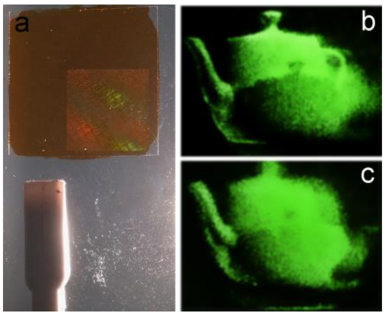

Supplementary Figure 6. Demonstration of the size up-scalable rGO holograms for 3D images with sufficient high resolution. a, One example of rGO holograms with 8000 by 8000 pixels. The size of the hologram is on the centimeter scale. The acquired images when focused on the back (b) and front (c) of the two teapots. High resolution images of more than 5,000 dpi can be obtained, corresponding to two-order of magnitude higher than that of the state-of-the-art of write-once metamaterial [1-3] and carbon nanotube [14] holograms.

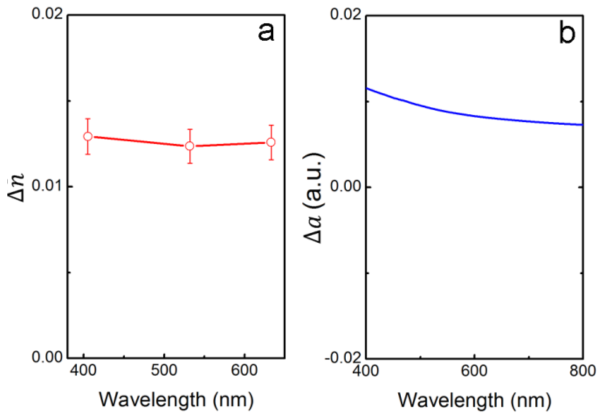

Supplementary Figure 7. a, Measured refractive-index modulation of the photoreduction of GO-polymers at three wavelengths of 405, 532 and 632 nm, respectively. b, localized absorbance change in the visible range after the photoreduction.

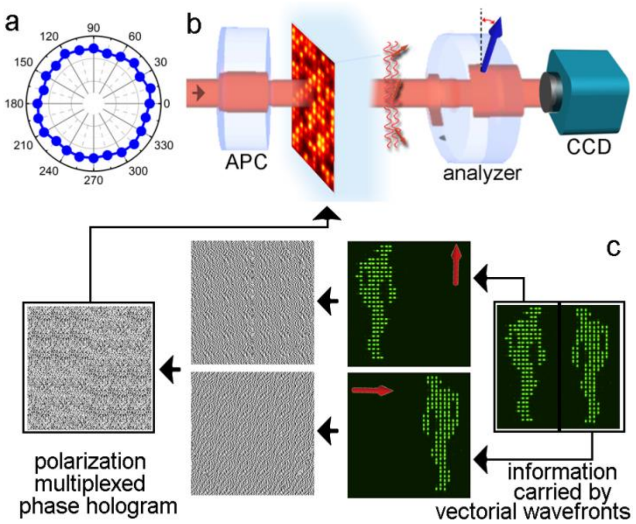

Supplementary Figure 8. a, The isotropic/polarization-insensitive refractive-index modulation of rGOs was confirmed by the microscopic characterization under linearly polarized illumination with the variation in the polarization orientation. b, Schematic illustration of the experimental configuration of the reconstruction of vectorial wavefronts. The polarization-sensitive information carried by the vectorial wavefront can be clearly discerned by rotating the polarization angle (indicated by the blue arrow) of the analyzer in front of the CCD camera. APC represents the azimuthal polarization converter. The red arrows schematically illustrate spatially variant polarization orientations in a 3D wavefront. c, Illustration of the principle of generating polarization-multiplexed phase holograms for the vectorial reconstruction of polarization discernible images.

Supplementary discussions

Refractive-index change by the athermal photoreduciton through a single fs pulse. In the previous studies, a high-temperature deoxygenation process by a constant thermal treatment [15-18] is essential to vitalize their electronic properties through producing a contrast in the imaginary part of the complex refractive index or conductivity ($\sigma(\omega)$) of GOs as [19]

$$\tilde{n}(\omega) = n(\omega) + j\frac{\sigma(\omega)}{2n(\omega)\omega} \qquad (2)$$

where $n(\omega)$ and $\sigma(\omega)$ are real part of refractive index and conductivity, respectively, at a given frequency ω. Since the focal temperature increase has been ruled out through the exposure to a single pulse from the temperature measurement, the thermally activated deoxygenation process can then be excluded from the reduction process of GOs. The temperature-free or athermal nature of the observed reduction can be possibly attributed to the photoionization process by absorbing the single pulse, which is likely responsible for the observed sub-wavelength scale contrast in the real part of the refractive index ($n(\omega)$), and hence allowing for writing holographically-correlated phase modulation. The athermal nature enables to confine the induced refractive-index modulation to a sub-wavelength scale by increasing the NA of the objective lens, which is impossible when the photoreduction associated with the thermal treatment induced by pulse trains operating at 80 MHz is employed [20].

Supplementary methods

Parallel optical digitalization using the Debye-diffraction method. Controlling the phase of the impinging femtosecond (fs) pulsed beam at the back aperture of the objective, we can generate a diffraction-limited MMA which can be used for parallelly digitalizing the phase modulation in the focal plane. A fs pulsed laser beam (Spitfire, a pulse duration of 100 fs and a repetition rate of 1KHz) at the wavelength of 800 nm was modulated with the Debye-diffraction method to take the depolarization effect into consideration as [10,21]

$$I_n = |E_{nx}|^2 + |E_{ny}|^2 + |E_{nz}|^2 \qquad (1)$$

where I_n is the intensity of the n_{th} focal spot and E_{nx}, E_{ny} and E_{nz} represent the three orthogonal field components by the Debye-diffraction theory, respectively. The phase distribution at the back aperture of the objective was retrieved by using the iterative algorithm to calculate the multifocal array according to the digitalized intensities in the focal plane. The generated multifocal spots in the array are all diffraction-limited and their intensities can be individually controlled for parallel digitization. Limited by the maximum power of our system, the parallel digitalization was configured to a MMA composed of 20 by 20 focal spots.

Supplementary References

1 Larouche, S., Tsai, Y.-J., Tyler, T., Jokerst, N. M. & Smith, D. R. Infrared metamaterial phase holograms. *Nat. Mater.* **11**, 450-454 (2012).

2 Ni, X., Kildishev, A. V. & Shalaev, V. M. Metasurface holograms for visible light. *Nat. Commun.* **4**, 2807 (2013).

3 Huang, L., Chen, X., Mühlenbernd, H., Zhang, H., Chen, S., Bai, B., Tan, Q., Jin, G., Cheah, K.-W., Qiu, C.-W., Li, J., Zentgraf, T. & Zhang, S. Three-dimensional optical holography using a plasmonic metasurface. *Nat. Commun.* **4**, 2808 (2013).

4 Ferrari, A. C. Raman spectroscopy of graphene and graphite: Disorder, electron-phonon coupling, doping and nonadiabatic effects. *Solid State Commun.* **143**, 47-57 (2007).

5 Maestro, L. M., Rodríguez, E. M., Rodríguez, F. S., La Cruz, M. C. I. D., Juarranz, A., Naccache, R., Vetrone, F., Jaque, D., Capobianco, J. A. & Solé, J. G. CdSe quantum dots for two-photon fluorescence thermal imaging. *Nano Lett.* **10**, 5109-5115 (2010).

6 Wu, X., Sprinkle, M., Li, X., Ming, F., Berger, C. & De Heer, W. A. Epitaxial-graphene/graphene-oxide junction: An essential step towards epitaxial graphene electronics. *Phys. Rev. Lett.* **101**, 026801 (2008).

7 Li, X., Lan, T. H., Tien, C. H. & Gu, M. Three-dimensional orientation-unlimited polarization encryption by a single optically configured vectorial beam. *Nat. Commun.* **3**, 998 (2012).

8 Ullah, M. A., Li, X., Cheng, X., Hao, X., Su, Y., Ma, J. & Gu, M. Low energy-density recording with a high-repetition-rate laser beam in gold-nanorod-embedded discs. *Opt. Express.* **20**, 24516-24523 (2012).

9 Zhou, Y., Bao, Q., Varghese, B., Tang, L. A. L., Tan, C. K., Sow, C. H. & Loh, K. P. Microstructuring of graphene oxide nanosheets using direct laser writing. *Adv. Mater.* **22**, 67-71 (2010).

10 Lin, H., Jia, B. & Gu, M. Dynamic generation of Debye diffraction-limited multifocal arrays for direct laser printing nanofabrication. *Opt. Lett.* **36**, 406-408 (2011).

11 Gu, M., Lin, H. & Li, X. Parallel multiphoton microscopy with cylindrically polarized multifocal arrays. *Opt. Lett.* **38**, 3627-3630 (2013).

12 Ren, H., Lin, H., Li, X. & Gu, M. Three-dimensional parallel recording with a Debye diffraction-limited and aberration-free volumetric multifocal array. *Opt. Lett.* **39**, 1621-1624 (2014).

13 Jia, B., Serbin, J., Kim, H., Lee, B., Li, J. & Gu, M. Use of two-photon polymerization for continuous gray-level encoding of diffractive optical elements. *Appl. Phys. Lett.* **90**, 073503 (2007).

14 Butt, H., Montelongo, Y., Butler, T., Rajesekharan, R., Dai, Q., Shiva-Reddy, S. G., Wilkinson, T. D. & Amaratunga, G. A. J. Carbon nanotube based high resolution holograms. *Adv. Mater.* **24**, OP331-OP336 (2012).

15 Wei, Z., Wang, D., Kim, S., Kim, S. Y., Hu, Y., Yakes, M. K., Laracuente, A. R., Dai, Z., Marder, S. R., Berger, C., King, W. P., De Heer, W. A., Sheehan, P. E. & Riedo, E. Nanoscale tunable reduction of graphene oxide for graphene electronics. *Science* **328**, 1373-1376 (2010).

16 Zhang, K., Fu, Q., Pan, N., Yu, X., Liu, J., Luo, Y., Wang, X., Yang, J. & Hou, J. Direct writing of electronic devices on graphene oxide by catalytic scanning probe lithography. *Nat. Commun.* **3**, 1194 (2012).

17 El-Kady, M. F. & Kaner, R. B. Scalable fabrication of high-power graphene micro-supercapacitors for flexible and on-chip energy storage. *Nat. Commun.* **4**, 1475 (2013).

18 Gao, W., Singh, N., Song, L., Liu, Z., Reddy, A. L. M., Ci, L., Vajtai, R., Zhang, Q., Wei, B. & Ajayan, P. M. Direct laser writing of micro-supercapacitors on hydrated graphite oxide films. *Nat. Nanotechnol.* **6**, 496-500 (2011).

19 Seybold, J. S. *Introduction to RF Propagation*. (John Wiley & Sons, 2005).

20 Li, X., Zhang, Q., Chen, X. & Gu, M. Giant refractive-index modulation by two-photon reduction of fluorescent graphene oxides for multimode optical recording. *Sci. Rep.* **3**, 2819 (2013).

21 Gu, M. *Advanced optical imaging theory*. (Springer, 2000).
